# Free-Propagator Reweighting Integrator for Single-Particle Dynamics in Reaction-Diffusion Models of Heterogeneous Protein-Protein Interaction Systems

**DOI:** 10.1103/PhysRevX.4.031037

**Published:** 2014-09-04

**Authors:** Margaret E. Johnson, Gerhard Hummer

**Affiliations:** 1 Department of Biophysics, The Johns Hopkins University, Baltimore, Maryland 21218, USA; 2 Department of Theoretical Biophysics, Max Planck Institute of Biophysics, Max-von-Laue Strasse 3, 60438 Frankfurt am Main, Germany

## Abstract

We present a new algorithm for simulating reaction-diffusion equations at single-particle resolution. Our algorithm is designed to be both accurate and simple to implement, and to be applicable to large and heterogeneous systems, including those arising in systems biology applications. We combine the use of the exact Green's function for a pair of reacting particles with the approximate free-diffusion propagator for position updates to particles. Trajectory reweighting in our free-propagator reweighting (FPR) method recovers the exact association rates for a pair of interacting particles at all times. FPR simulations of many-body systems accurately reproduce the theoretically known dynamic behavior for a variety of different reaction types. FPR does not suffer from the loss of efficiency common to other path-reweighting schemes, first, because corrections apply only in the immediate vicinity of reacting particles and, second, because by construction the average weight factor equals one upon leaving this reaction zone. FPR applications include the modeling of pathways and networks of protein-driven processes where reaction rates can vary widely and thousands of proteins may participate in the formation of large assemblies. With a limited amount of bookkeeping necessary to ensure proper association rates for each reactant pair, FPR can account for changes to reaction rates or diffusion constants as a result of reaction events. Importantly, FPR can also be extended to physical descriptions of protein interactions with long-range forces, as we demonstrate here for Coulombic interactions.

## I. Introduction

Proteins perform functions ranging from signal transmission and transcriptional regulation to assembly into structural scaffolds by stochastically binding with one another in the cellular environment. Approaches to modeling these processes at a scale capable of capturing the dynamics of whole populations of proteins vary in the degree of spatial resolution and the physical, empirical, or phenomenological rules describing the interactions between proteins. At perhaps the coarsest level, rate equations have long been used quite successfully to model large and relatively complex systems of reacting species that are assumed to be homogeneously distributed in space. However, the spatial distribution of proteins is never truly uniform throughout the cell, and the effect of spatial localization on protein dynamics is a critical factor in modeling phenomena such as pattern development in fly embryos [1] and activation of mitogen-activated protein kinase (MAPK) signaling pathways in response to pheromone stimulation [2,3]. Capturing protein dynamics in space and time also accounts for fluctuations in local protein concentrations that can, for example, modulate the speed and stability of signal pathway response [4]. Ultimately, spatial resolution is necessary to capture the structural and molecular details of proteins that underpin their specific interactions with one another.

Numerical simulation is necessary for solving these generally nonequilibrium many-body problems for all but the simplest systems. Theoretical approaches, including field-theoretic [5,6], Smoluchowski-type [7], and other analytical methods [8], can provide quantitative or exact descriptions of time-dependent behavior in specific and relatively simple systems that can be used to test the accuracy of numerical methods. The numerical solutions of the equations governing the spatial and temporal dynamics of reacting species can be broadly categorized into single-particle methods and concentration-based methods. Concentration-based methods describe the evolution of the protein concentrations as a result of both chemical reactions and diffusion using deterministic partial differential equations (PDE) or stochastic master equations, such as the reaction-diffusion master equation (RDME) [9]. For deterministic PDEs, the systems of generally nonlinear coupled equations are numerically solved by splitting the volume into voxels and propagating the equations using continuum finite element methods [3,10]. Numerical solutions to the RDME also partition space into subvolumes and update species concentrations using lattice spatial Gillespie-type algorithms [11,12]. These methods can reach large time and length scales and have been used for full-cell simulations [13] where detailed tracking of individual particles may be unimportant. An alternate mesoscopic dynamical scheme obeying the Navier-Stokes equation tracks the velocities of particles in spatial sub-volumes and accounts for both reactive and nonreactive collisions between species [14]. Single-particle methods, by contrast, offer high-resolution representations but are therefore more costly to implement at the same scale. However, they are necessary to accurately capture the behavior of systems where, for instance, receptor aggregation or ligand localization controls signal transduction [4,15,16]. Recently, hybrid methods have been developed to add single-particle resolution to specific regions of otherwise concentration-based models [17,18]. Single-particle approaches also allow one to expand the physical description of particle interactions from reaction-rate kinetics to incorporate distance-dependent forces between particles and orientational or rotational constraints that begin to connect to a molecular level representation of protein interaction dynamics in a multiscale representation. The free-propagator reweighting (FPR) algorithm we develop here treats the system at single-particle resolution and offers the flexibility to incorporate these more microscopic details into the description of protein-protein interactions.

A major challenge in single-particle methods is the accurate modeling of diverse types of reactions between different species within the same system, while still reaching relatively large length and time scales. The many-body problem of individual particles interacting is numerically solved by breaking up the system into a series of two-body problems, with different methods using different frameworks to define two-body reactions. Brute force Brownian dynamics (BD) can be used to accurately sample the many-body dynamics of particles diffusing and reacting on collisions, but it requires very short time steps (picosecond to nanosecond range), particularly as two particles come into close contact, and it is nontrivial to extend such direct simulations to reversible reactions. Recent approaches designed to lengthen the time step for BD simulations [19] do not reach the larger time steps needed to observe biological assembly processes. Several methods designed to reach a full cellular scale by taking larger time steps (microsecond to millisecond) replace reactive collisions with phenomenological probability models that are derived to reproduce, for example, the bulk reaction rate [20]. The dynamics of these rule-based numerical approaches, however, is not exactly governed by any PDEs and therefore the behavior of the system may not faithfully reproduce the behavior of BD simulations or results from concentration-based methods. The more quantitative approach to defining reactive and reversible collisions implemented in the SmolDyn package [15] reproduces the correct total on and off rates for individual bimolecular reactions [1,21,22]. However, in this method, the physical contribution to the total on and off rates from either the diffusion to contact or reaction on contact depends on the size of the time step. With larger time steps, the on rate of the reaction will be dominated by the contribution from the rate constant, whereas with shorter time steps, the on rate will be dominated by the contribution due to diffusion. As a result, changing the size of the time step will also change the type of dynamics obeyed by the interacting particles and the effective rate of unbinding and rebinding reactions. Because the reaction dynamics is not independent of the simulation parameters, this approach is not consistently providing a numerical solution to a specific set of differential equations. In practice, this would make it difficult to simultaneously capture accurate dynamics of the diffusion and reaction processes for diverse coupled systems of reaction rate-limited and diffusion-influenced reactions. A rigorous approach that does succeed in exactly reproducing the dynamics of diffusing and reacting particles in many-body systems is Green's function reaction diffusion (GFRD) [4,23]. The GFRD algorithm [4,23] and earlier approaches [24,25] use the Green's function (GF) solution to the PDE for a pair of particles diffusing with a reactive boundary between them to propagate particle positions and evaluate reaction probabilities.

The benefits of using GFs to propagate particles are not only the exact treatment at least of pairwise interactions, but also the ability to use large time steps while correctly accounting for different possible collision encounters. Whether particles always react on collision (absorbing boundary [7]) or react with a fixed-rate constant (radiation boundary conditions [26]), the probability of associating has a closed-form analytic solution that can be directly evaluated [27]. Building in forces between particles is possible through adding a drift term to the diffusion propagator, although the GF for this problem is only solvable numerically. The major disadvantage of using GFs is the difficulty in correctly sampling positions from the propagator. Particle positions do not update freely according to Brownian dynamics, but rather the separation between a pair of interacting particles must be sampled from the GF distribution. The GF distribution, while of closed form, is not invertible for the positions and therefore look-up tables must be generated for each reaction type. Because the GF is dependent on the reaction rate, the binding radius, the diffusion constant, the size of the time step, and the starting separation between the particles, this requires substantial precalculation and storage of GF solutions to run a simulation. Additionally, the positions are sampled from a GF solution that is a computationally costly infinite sum, which the FPR method developed here avoids. Finally, to ensure the exactness of the GFRD method for many interacting particles, the time step is selected at each step along with protective domains [28] for particles to partition the system into exclusively single or pairwise interactions. While the GFRD method is remarkable in its efficiency and accuracy in exactly solving a many-body problem, for systems with a large number of diverse reactants, it is costly to implement and run.

**Fig. 1:**
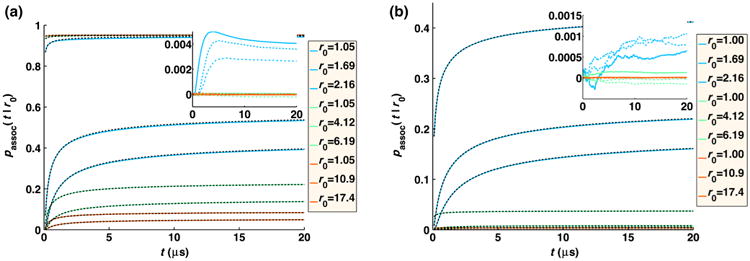
Probability of two particles having associated between times 0 and *t*, given they were separated by a distance *r*_0_ at *t* = 0. Results for the exact propagator are shown as black dashed lines [Eq. (12)], where the binding radius is *σ* = 1 nm. The probability of associating from FPR simulations is shown for *D_AB_* = 1 nm^2^/*μ*s (blue), *D_AB_* = 20 nm^2^/*μ*s (green), and *D_AB_* = 200 nm^2^/*μ*s (orange). (a) Results for perfectly absorbing boundary (*k_a_*=∞) and a time step of Δ*t* = 0.1 *μ*s. Simulations are averaged over 10^6^ trajectories each initialized to the prescribed *r*_0_. The inset plots the difference between exact and simulated results [*p*_exact_(*r*, *t*|*r*_0_) *– p*_sim_(r; *t*|*r*_0_)]. The simulated results marginally underestimate the association rate due to incomplete trajectory sampling, with errors being largest for slow diffusion. (b) Same as (a) but with radiation BCs at *σ* = 1 nm with *k_a_* = 10 nm^3^/*μ*s. For this lower rate (relative to absorbing where *k_a_* = ∞), the error is smaller for all *D_AB_*.

In the FPR approach, we combine the use of the GF to evaluate particle interactions with positions that are sampled from the much simpler free-diffusion propagator to produce a method that extends readily to diverse systems while still maintaining high accuracy, as we detail below. The simple position updates also means our method could readily be incorporated into existing BD software. The error we introduce into the dynamics, and, therefore, the association rates of the particles by using the free propagator, can be measured at each time step and corrected for using a simple trajectory reweighting approach. With this trajectory reweighting, our FPR method will recover the exact association rates between reactants at each time step, as shown in Fig. 1, relative to the exact solution for various reaction conditions. By sampling positions from the free propagator, we dramatically simplify the position updates, which only require selecting Gaussian random numbers with an extra step to ensure the particles do not overlap. The simplicity of the particle position updates means we do not have to precalculate or store any large arrays and, therefore, allows us to readily expand the systems to a variety of reaction types. Additionally, this allows us to adapt to changes to the diffusion constant that might occur with complex formation during the simulations, or changes to reaction rates. Evaluating the association probability given by the GF simply requires the evaluation of the function at a particular position, so no inversion or sampling is necessary. The sampling efficiency of the FPR reweighting approach is optimized by ensuring that the average reweighting ratio upon exit from a reaction zone equals one by construction. While the particle dynamics is only approximate when approaching a reactive partner, by reproducing the exact association rates, we show that the correct behavior of both pairwise and many-body systems is still recovered for reaction types ranging from the reaction-rate limited to the diffusion-limited regime.

In this paper, we first review the single-particle approach to modeling reactive species using the GF. We then show that, by using the free propagator with appropriate trajectory reweighting, the exact association rates will be recovered. We detail the implementation of this FPR method for propagating many-body systems of particles to efficiently and accurately model heterogeneous populations of reactive species. Furthermore, we demonstrate the application of the FPR method to species that not only react upon collisions but also interact through a distance-dependent pair potential. This represents an important advancement for building more accurate physics into the protein-protein interactions. We note some issues that can occur when trajectories are undersampled as well as some additional considerations arising from long-range potentials and we present approaches to deal with these. Using several examples of reaction types for diffusion-limited, diffusion-influenced, and reaction-rate-limited association, we compare FPR simulation results with available theoretical descriptions of both the equilibrium and time-dependent relaxation behavior of the systems. While the FPR method does not provide the exact solution for particle pairs of the GFRD method [4,23], it is, nonetheless, highly accurate, as our results show, and is relatively simple, efficient, and flexible to implement even in complex systems with many components and reactions. Finally, we discuss the general approach of trajectory reweighting as applicable to GF methods beyond those studied here and opportunities for high-resolution modeling of new biological phenomena.

## II. Theory

### A. Background and notation

The motions of a system of particles in solution can be approximated as Brownian dynamics described by the Smoluchowski diffusion equation. Binding processes or chemical reactions between colliding particles can be added to this Fokker-Planck–type PDE by imposing boundary conditions at a specified contact distance for each reactant pair. The general PDE for *N* particles with Cartesian positions ***r⃗*** ≡ (***r****_A_*, ***r****_B_*, …, ***r****_N_*)*^T^* = (*x_A_*, *y_A_*, *z_A_*, …, *x_N_*, *y_N_*, *z_N_*)*^T^* that interact according to a potential *V*(***r⃗***) and/or react with one another is a many-body problem that cannot be solved analytically in general. For such particles with initial positions *r⃗*_0_, the probability density of their positions being ***r⃗*** at time *t* satisfies

(1) $$\frac{\partial p(\vec{r},t|{\vec{r}}_{0})}{\partial t}=\frac{\partial^{T}}{\partial \vec{r}}D(\vec{r})e^{-\beta V(\vec{r})}\frac{\partial }{\partial \vec{r}}e^{+\beta V(\vec{r})}p(\vec{r},t|{\vec{r}}_{0}),$$

where the diffusion tensor ***D***(***r⃗***) is a positive-definite symmetric matrix that is diagonal for particles undergoing isotropic diffusion and constant for position-independent diffusion, and *β* is the inverse temperature.

We approach this many-body problem by breaking it up into individual pairwise interactions to revert to a series of tractable two-body problems. For a single pair of particles *A* and *B*, with interactions and reactions dependent only on their relative distance *r* = *r_AB_*, Eq. (1) reduces to one spatial dimension and is analytically solvable for reactive systems without interaction potential, *V* = 0. In Sec. II C, we consider nonzero interaction potentials. Here, we focus on spherically symmetric reactions that depend on the radial distance *r* between the particles, resulting in the PDE (in spherical coordinates)

(2) $$\frac{\partial p(r,t|r_{0})}{\partial t}=D(\frac{\partial }{\partial r}+\frac{2}{r})\frac{\partial }{\partial r}p(r,t|r_{0}),$$

where *D* = *D_AB_* = *D_A_* + *D_B_* is the total diffusion constant and *r*_0_ is the initial separation at *t* = 0. Here and in the following, *D_A_* = *D_B_*, unless noted otherwise, and radial distribution functions are normalized to $\int_{0}^{\infty }4\pi r^{2}p(r,t|r_{0})=1$, i.e., with a Jacobian factor of 4*πr*^2^. The initial condition is simply a delta function at the starting position

(3) $$p(r,0|r_{0})=\delta (r-r_{0})/4\pi r_{0}^{2}.$$

In an infinite volume, the first boundary condition (BC) is given by

(4) p(r→∞,t)=0.

For particles that do not react with one another, this is the only BC and the solution to Eq. (2) is the GF for the free propagator:

(5) $$p_{\text{free}}(r,t|r_{0})=\frac{1}{4\pi rr_{0}\sqrt{D}}\frac{1}{\sqrt{4\pi t}}[\exp(-\frac{{\left(r-r_{0}\right)}^{2}}{4Dt})-\exp(-\frac{{\left(r+r_{0}\right)}^{2}}{4Dt})].$$

For particle pairs that react with a rate coefficient *k_a_* at contact (*r* = *σ*), a second BC is

(6) $$4\pi \sigma^{2}D\frac{\partial p(r,t|r_{0})}{\partial r}{|}_{r=\sigma }=k_{a}p(\sigma ,t|r_{0}).$$

This radiation BC becomes reflecting for *k_a_* = 0. For *k_a_* = ∞, the BC is replaced by the absorbing BC, *p*(*σ*, *t*|*r*_0_) = 0, where all particles at contact associate. *k_a_* specifically defines the association rate for particles at contact, and is therefore not necessarily identical to the apparent experimental rate *k*_on_, defined in Sec. IV A, which also accounts for diffusion to contact. The solution to Eq. (2) that accounts for the radiation boundary condition [Eq. (6)] is the GF

(7) $$p_{\text{irr}}(r,t|r_{0})=\frac{1}{4\pi rr_{0}\sqrt{D}}\{\frac{1}{\sqrt{4\pi t}}[\exp(-\frac{{\left(r-r_{0}\right)}^{2}}{4Dt})+\exp(-\frac{{\left(r+r_{0}-2\sigma \right)}^{2}}{4Dt})]-\alpha \exp(2\alpha \frac{r+r_{0}-2\sigma }{\sqrt{4D}}+\alpha^{2}t)\times \text{erfc}(\frac{r+r_{0}-2\sigma }{\sqrt{4Dt}}+\alpha \sqrt{t})\},$$

where *k_D_* = 4*πσD* and *α* = (√*D*/*σ*)(1 + (*k_a_*/*k_D_*)). The distribution *p*_irr_ of particle pair distances accounts for irreversible association at contact. The reversible GF that also accounts for particles dissociating to contact is derived in Ref. [29]. The implementation of reversibly reacting particles will be discussed further below, but is accurately implemented using the irreversible association probability and a Poissonian dissociation process.

The volume integral over the GF distribution defines the probability that the two particles have survived to time *t* without reacting:

(8) $$S(t|r_{0})=\int_{\sigma }^{\infty }dr4\pi r^{2}p_{\text{irr}}(r,t|r_{0}).$$

The complement of this survival probability then defines the probability of having associated in a time Δ*t* given a starting position at *r*_0_:

(9) $$p_{\text{assoc}}(t|r_{0}\equiv 1-S(t|r_{0})=\frac{\sigma }{r_{0}}\frac{k_{a}}{k_{a}+k_{D}}[\text{erfc}(\frac{r_{0}-\sigma }{\sqrt{4Dt}})-\exp(2\alpha \frac{r_{0}-\sigma }{\sqrt{4D}}+\alpha^{2}t)\times \text{erfc}(\frac{r_{0}-\sigma }{\sqrt{4Dt}}+\alpha \sqrt{t})].$$

These definitions will be used in the methods below.

We note that to use the free propagator to sample the positions of each particle while still enforcing a reactive boundary at *σ*, the distribution of separations between particles excluded from the volume defined by *r* < *σ* must firstly be renormalized to give:

(10) $$p_{\text{free}}^{\text{norm}}(r,t|r_{0})=\frac{p_{\text{free}}(r,t|r_{0})}{\int_{\sigma }^{\infty }p_{\text{free}}(r,t|r_{0})4\pi r^{2}dr}.$$

This is because the free propagator is derived to sample all separations down to *r* = 0, but we will reject and redraw all moves that sample *r* < *σ*, as explained in Sec. III A. Secondly, if the particles react with one another, then the probability of any specific separation will be rescaled to account for some particles having associated rather than diffused from the previous position. Therefore, the distribution is rescaled by the exact survival probability to give

(11) $$p_{\text{free}}^{*}(r,t|r_{0})=\frac{p_{\text{free}}(r,t|r_{0})}{\int_{\sigma }^{\infty }p_{\text{free}}(r,t|r_{0})4\pi r^{2}dr}S(t|r_{0}).$$

We note here that we will be performing trajectory reweighting, and this procedure affects the survival probability at each time step. When trajectory reweighting is applied, the value of the survival probability at each time step is one minus the *reweighted* association probability, which changes it from the closed-form solution in Eq. (9). A ramification of these necessary adjustments to the free propagator to perform reactions and trajectory reweighting is that the probability of a multistep trajectory sampled with trajectory reweighting does not have a simple analytic expression, since the reweighting ratios at each time step must be kept track of.

### B. Free-propagator trajectory reweighting

To correctly describe the dynamics for two interacting particles as prescribed by the distribution in Eq. (7), one must associate particles in a given time step according to Eq. (9) and otherwise sample the separation of the particle pairs using Eq. (7). This is what is done in the GFRD algorithm developed by van Zon and ten Wolde [23,30]. However, sampling from Eq. (7), which is effectively 1D in the radial separation, will not fully determine 3D positions of the particles. To describe the full 3D position dependence of particles obeying Eq. (7) requires sampling from the full 3D version of the GF [30]. Because this sampling requires the definition of 3D look-up tables for each reaction type and for each value of the current separation *r*_0_, we propose an alternative approach. However, the 3D propagator can also be constructed from a truncated expansion in precalculated Bessel functions, as implemented in the software [4].

**Fig. 2:**
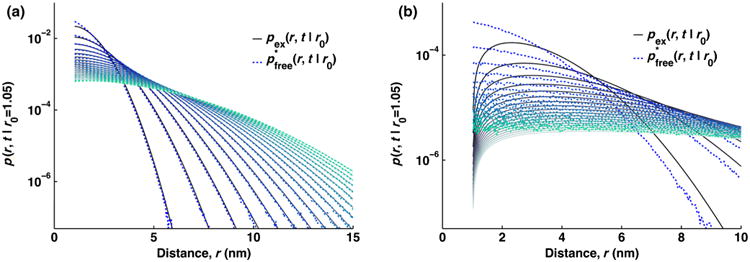
Green's function for a pair of particles being propagated using Eq. (7) (dark solid lines) and propagated using Eq. (11) (blue dashed lines) with trajectory reweighting. Each curve represents the particle distribution after another time step, top to bottom at *r* = *σ*, with particles initially placed close to contact at *r*_0_ = *σ +* 0.05 nm. (a) *k_a_* =0.1 nm^3^/*μ*s, *D_AB_* = 5 nm^2^/*μ*s, and Δ*t* = 0.1 *μs*. (b) *k_a_* = ∞, *D_AB_* = 20 nm^2^/*μ*s, and Δ*t* = 0.1 *μs*. The deviations between the exact and the free propagators varies depending on the reaction parameters. The FPR algorithm uses trajectory reweighting to recover the association rates that would result if positions were sampled using the exact propagator.

Specifically, we seek to simplify the sampling of particle positions while still ensuring that the correct rates of association are preserved. Instead of sampling particle positions from the 3D version of Eq. (7), we sample positions from the free propagator, Eq. (5). Sampling 3D positions according to the free propagator [Eq. (5)] is much simpler. It amounts to adding independent Gaussian random numbers of mean zero and standard deviation $\sqrt{2D_{A}\Delta t}$ to each of the three Cartesian coordinates of a particle with diffusion coefficient *D_A_*. The two distributions sampled by the free propagator and the reactive propagator are, in general, different until the particles move away from the reactive boundary (*r* ≫ *σ*), where the dynamics is accurately described by free diffusion [Eq. (5)]. In Fig. 2, we compare the propagator for the exact irreversible association with the propagator that uses the free-diffusion distribution to sample positions. At short times and close distances, we find the expected differences between the exact and simulated positional distributions; at longer times and separations, these differences diminish because the overall association rate is essentially exact. As shown below, the correct rates and equilibrium can thus be recovered by reweighting the association probability by the trajectory probabilities. Hence, although the dynamics of position updates will only be approximate at short separations, the rate of association will be exact. At larger separations, the dynamics becomes exact as well.

The quantity that we seek to correctly reproduce despite sampling positions from the incorrect propagator [Eq. (5) instead of Eq. (7)] is the probability of associating in a time interval Δ*t* given a current time *T* and an initial separation of *r*_0_ at time *t* = 0. The exact version of this quantity can be written as

(12) $$p_{\text{assoc}}(T+\Delta t|r_{0})=\int_{\sigma }^{\infty }4\pi r^{2}drp_{\text{irr}}(r,T|r_{0})p_{\text{assoc}}(\Delta t|r),$$

where *p*_irr_ and *p*_assoc_ are defined by Eqs. (7) and (9), respectively. For *T* = 0, *p*_irr_ is the initial condition in Eq. (3) and identity is recovered. For *T* > 0 *p*_irr_ accounts for the passage of time from *t* = 0 to *t* = *T*. In the final time step Δ*t*, we then must average over all possible end points that would occur as a result of all trajectories of length *T*.

The association probability in Eq. (12) can be rewritten by first breaking up the probability *p*_irr_(*r*, *T*|*r*_0_) of trajectories that travel from *r*_0_ at *t* = 0 to *r* at *t* = *T* into a series of discrete steps. By integrating all possible paths through these discrete time points, the probability of trajectories can be written as

(13) $$p_{\text{irr}}(r,T|r_{0})=\int_{\sigma }^{\infty }d\Omega_{\Delta t}d\Omega_{2\Delta t}\cdots d\Omega_{T}p_{\text{irr}}(r_{T},T;r_{T-\Delta t},T-\Delta t;\ldots ;r_{\Delta t},\Delta t|r_{0}),$$

and accordingly, Eq. (12) becomes

(14) $$p_{\text{assoc}}(T+\Delta t|r_{0})=\int_{\sigma }^{\infty }d\Omega_{\Delta t}d\Omega_{2\Delta t}\cdots d\Omega_{T}p_{\text{irr}}(r_{T},T;r_{T-\Delta t},T-\Delta t;\ldots ;r_{\Delta t},\Delta t|r_{0})p_{\text{assoc}}(\Delta t|r_{T}),$$

where we use the notation $d\Omega_{t}=4\pi r_{t}^{2}dr$ to indicate the volume integral performed at each time point *t* along the trajectory. Because of the Markov property, we can factorize the path probability,

(15) $$p_{\text{irr}}(r_{T},T;r_{T-\Delta t},T-\Delta t;\ldots ;r_{\Delta t},\Delta t|r_{0})=p_{\text{irr}}(r_{T},T|r_{T-\Delta t},T-\Delta t)\cdots p_{\text{irr}}(r,\Delta t|r_{0}).$$

This property is critical to the implementation of the algorithm, as the probability of the current trajectory is just the accumulated product of all earlier steps.

Finally, Eq. (12) becomes

(16) $$p_{\text{assoc}}(T+\Delta t|r_{0})=\int_{\sigma }^{\infty }d\Omega_{\Delta t}d\Omega_{2\Delta t}\cdots d\Omega_{T}p_{\text{irr}}(r_{T},T|r_{T-\Delta t},T-\Delta t)\cdots p_{\text{irr}}(r,\Delta t|r_{0})p_{\text{assoc}}(\Delta t|r_{T}).$$

This association probability clearly depends on the propagator, even if the correct value of *p*_assoc_(Δ*t*|*r_T_*) [Eq. (9)] is used. If we simply propagate using the free propagator, the approximate association rate is given by, via the same derivation,

(17) $$p_{\text{assoc}}^{\text{approx}}(T+\Delta t|r_{0})=\int_{\sigma }^{\infty }d\Omega_{\Delta t}d\Omega_{2\Delta t}\cdots d\Omega_{T}p_{\text{free}}^{*}(r,T|r,T-\Delta t)\cdots p_{\text{free}}^{*}(r,\Delta t|r_{0})p_{\text{assoc}}(\Delta t|r_{T}).$$

To recover the correct quantity defined by Eq. (16), we rescale the value of *p*_assoc_(Δ*t*|*r_T_*) at each time step by the ratio. $w_{\text{ratio}}=p_{\text{irr}}(r,\Delta t|r_{0})/p_{\text{free}}^{*}(r,\Delta t|r_{0})$. This ratio reflects the probability that this particular trajectory is sampled by the exact propagator relative to the probability it was sampled by the simulated free propagator. By factorizing the reweighting factor in terms of the ratio of trajectory probability changes along the pathway, the association rate at each time step changes to

(18) $$p_{\text{assoc}}^{\text{rewgt}}(\Delta t|r_{T})=p_{\text{assoc}}(\Delta t|r_{T})\frac{p_{\text{irr}}(r_{T},T|r_{T-\Delta t},T-\Delta t)p_{\text{irr}}(r_{T-\Delta t},T-\Delta t|r_{T-2\Delta t},T-2\Delta t)\cdots p_{\text{irr}}(r_{\Delta t},\Delta t|r_{0})}{p_{\text{free}}^{*}(r_{T},T|r_{T-\Delta t},T-\Delta t)p_{\text{free}}^{*}(r_{T-\Delta t},T-\Delta t|r_{T-2\Delta t},T-2\Delta t)\cdots p_{\text{free}}^{*}(r_{\Delta t},\Delta t|r_{0})}.$$

Inserting this reweighted association probability into Eq. (17) to replace *p*_assoc_(Δ*t*|*r_T_*) gives the final result:

(19) $$p_{\text{assoc}}^{\text{rewgt}}(T+\Delta t|r_{0})=\int_{\sigma }^{\infty }d\Omega_{\Delta t}d\Omega_{2\Delta t}\cdots d\Omega_{T}p_{\text{free}}^{*}(r_{T},T|r_{T-\Delta t},T-\Delta t)\cdots p_{\text{free}}^{*}(r_{\Delta t},\Delta t|r_{0})p_{\text{assoc}}^{\text{rewgt}}(\Delta t|r_{T})=\int_{\sigma }^{\infty }d\Omega_{\Delta t}d\Omega_{2\Delta t}\cdots d\Omega_{T}p_{\text{free}}^{*}(r_{T},T|r_{T-\Delta t},T-\Delta t)\cdots p_{\text{free}}^{*}(r_{\Delta t},\Delta t|r_{0})p_{\text{assoc}}(\Delta t|r_{T})\times \frac{p_{\text{irr}}(r_{T},T|r_{T-\Delta t}.T-\Delta t)\cdots p_{\text{irr}}(r_{\Delta t},\Delta t|r_{0})}{p_{\text{free}}^{*}(r_{T},T|r_{T-\Delta t},T-\Delta t)\cdots p_{\text{free}}^{*}(r_{\Delta t},\Delta t|r_{0})}=p_{\text{assoc}}(T+\Delta t|r_{0}),$$

thus, exactly recovering Eq. (16) for the overall probability of association after reweighting.

In Fig. 1, we compare the association rate as a function of time for two particles calculated using the exact propagator [Eq. (12)] and simulated numerically with the FPR trajectory reweighting method [Eq. (19)] and see excellent agreement. By contrast, simply using the free propagator without the trajectory reweighting results in too much association for large intrinsic rates (*k_a_*/*σ*^3^ = 1000 s^−1^), and too little association for small rates (*k_a_*/*σ*^3^ = 0.01 s^−1^) or small time steps (1 ns) (data not shown). The use of trajectory reweighting corrects for both under- and over-sampling of association that would otherwise result from using free propagators.

#### Trajectory overlap and reweighting zone

Although Eq. (19) reduces exactly back to Eq. (16) mathematically, an important caveat is that a trajectory must first be sampled before it can be reweighted. If the trajectory $p_{\text{free}}^{*}(r_{T},T;r_{T-\Delta t},T-\Delta t;\ldots ;r_{\Delta t},\Delta t|r_{0})$ has a vanishingly small probability, it will not normally be sampled, leading to an undersampling error if the actual path probability *p*_irr_(*r_T_*, *T*; *r*_*T*−Δ*t*_, *T*−Δ*t*; …;*r*_Δ*t*_, Δ*t*|*r*_0_) is significant. Sampling becomes an issue for long trajectories, where the differences in weight between the propagators accumulates and results in diminishing overlap in sampled trajectories versus exact trajectories. However, in most cases, this lack of overlap is not an issue because trajectory reweighting need only occur when the particles are within a reaction zone where they have the potential to associate, and therefore, trajectory probabilities need not be evaluated over long periods of time.

**Fig. 3:**
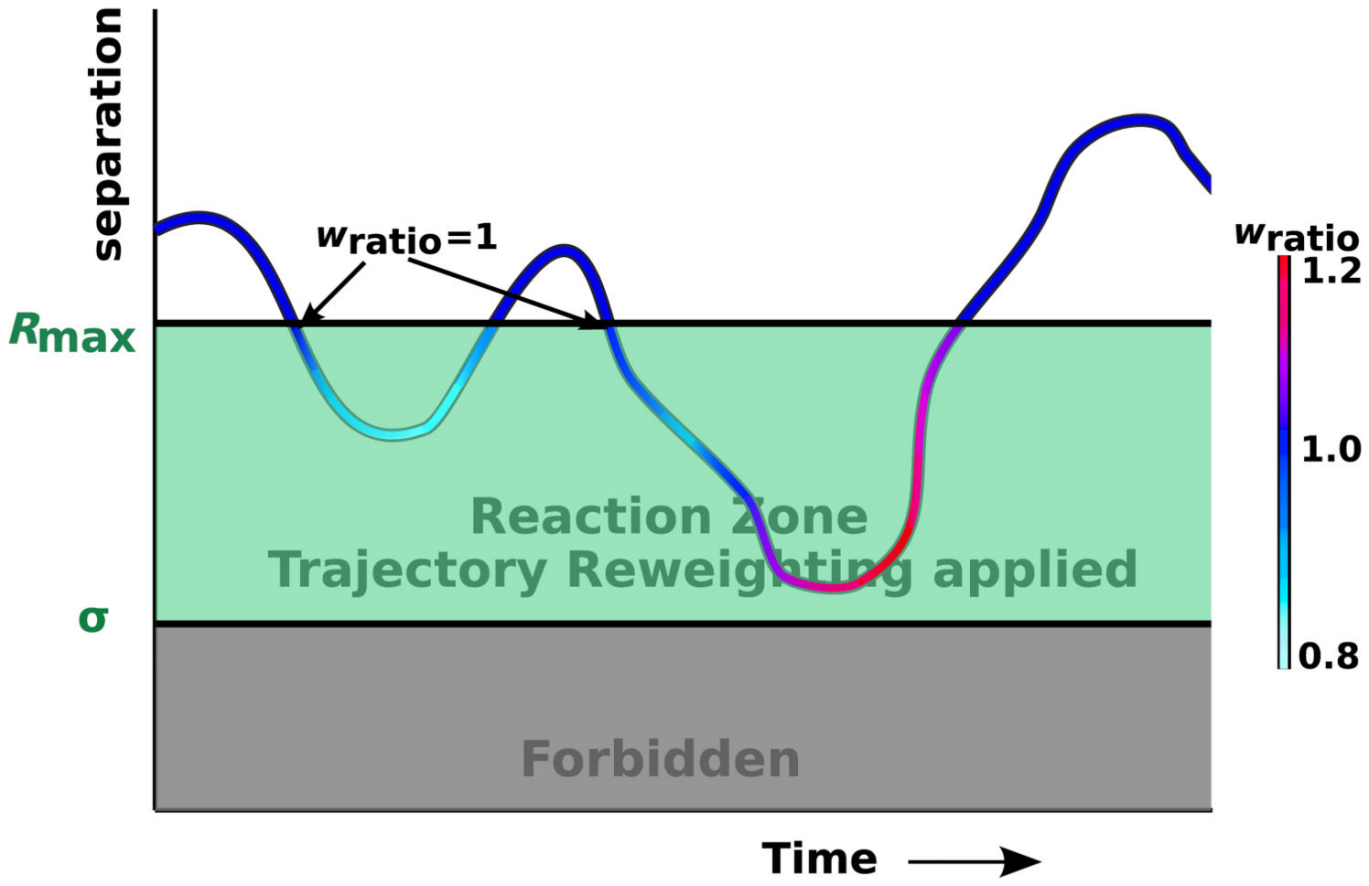
Illustration of a trajectory tracking the separation between a pair of particles versus time. The particles cannot come closer together than the binding radius *σ*, and when they are separated by less than *R*_max_, they have a nonzero probability of associating during the next time step, which increases the closer they get. Within the reaction zone, the probabilities of sampling this trajectory using the free-diffusion propagator and the exact RD propagator differ. Therefore, in this region, the association probability is reweighted by the ratio of trajectory probabilities to recover the correct association rate that would be achieved by using the exact propagator. The color scale indicates a possible example of how the reweighting ratio would evolve within the reaction zone. When the trajectory exits the reaction zone, its value of *w*_ratio_ will, in general, not be one, but because the average over all trajectories is one, and memory of the near encounter is lost, we can reinitialize *w*_ratio_ to one upon reentering the reaction zone.

By limiting the spatial range over which trajectory probabilities are calculated, we reduce the time length of sampled trajectories that are reweighted. This is important because the number of possible trajectory paths that could be sampled increases drastically with the length of simulation time and could lead to poor overlap with the exact distribution. We use a pair distance cutoff *R*_max_ to define a reaction zone, in which reweighting is applied to account for the nonzero probability of encounter and association during a time step. Starting from outside of this reaction zone, two particles have a vanishingly small chance to collide with one another during a time step Δ*t*, and the probability of associating drops to zero. Also, the free propagator correctly describes particle displacements here and therefore the value of *w*_ratio_ for the step is exactly one. *R*_max_ would, therefore, be defined as the separation where *p*_assoc_(Δ*t*|*R*_max_) [Eq. (9)] reaches zero or nearly zero. Altogether this results in an adjustment to Eq. (19) that limits reweighting to within the reaction zone defined by *R*_max_, such that Eq. (19) becomes

(20) $$p_{\text{assoc}}^{\text{rewgt}}(T+\Delta t|r_{0})=\int_{\sigma }^{R_{\max}}d\Omega_{\Delta t}d\Omega_{2\Delta t}\cdots d\Omega_{T}p_{\text{free}}^{*}(r_{T},T|r_{T-\Delta t},T-\Delta t)\ldots p_{\text{free}}^{*}(r_{\Delta t},\Delta t|r_{0})p_{\text{assoc}}(\Delta t|r_{T})\times \frac{p_{\text{irr}}(r_{T},T|r_{T-\Delta t},T-\Delta t)\ldots p_{\text{irr}}(r_{\Delta t},\Delta t|r_{0})}{p_{\text{free}}^{*}(r_{T},T|r_{T-\Delta t},T-\Delta t)\ldots p_{\text{free}}^{*}(r_{\Delta t},\Delta t|r_{0})}+\sum_{i=1}^{T}\int_{\sigma }^{R_{\text{max}}}d\Omega_{(i+1)\Delta t}\ldots d\Omega_{T}p_{\text{free}}^{*}(r_{T},T|r_{T},T-\Delta t)\ldots \times p_{\text{free}}^{*}(r_{(i+1)\Delta t},(i+1)\Delta t|R_{\max}^{-},i\Delta t)p_{\text{assoc}}(\Delta t|r_{T})\times \frac{p_{\text{irr}}(r_{T},T|r_{T-\Delta t},T-\Delta t)\ldots p_{\text{irr}}(r_{(i+1)\Delta t},(i+1)\Delta t|R_{\max}^{-},i\Delta t)}{p_{\text{free}}^{*}(r_{T},T|r_{T-\Delta t},T-\Delta t)\ldots p_{\text{free}}^{*}(r_{(i+1)\Delta t},(i+1)\Delta t|R_{\max}^{-},i\Delta t)}p_{\text{irr}}^{*}(R_{\max}^{-},t=i\Delta t|r_{0}),$$

where the “−” superscript indicates that the pair has approached the reaction zone from outside *R*_max_, and the * indicates that the trajectory may have passed in and out of the reaction zone without associating. The first integral on the right-hand side accounts for association from particles that start in the reaction zone (*r*_0_ < *R*_max_) and stay there throughout the time interval *T*. The second sum accounts for association from particles that (1) started outside the reaction zone and therefore could not associate until they entered the zone after at least a step or more, and (2) started inside the reaction zone, exited without associating, and reentered the reaction zone. With complete sampling, the trajectory probability $p_{\text{irr}}^{*}(r,t|r_{0})$ will be equal to *p*_irr_(*r*, *t*|*r*_0_) because trajectories that enter the reaction zone and exit before reacting will average to have the same probability under the FPR propagator as the exact irreversible propagator, as we discuss further below. Equation (20) states that all trajectories that start from *r*_0_ and stay in the reaction zone will contribute to association, as will all trajectories that enter or reenter the reaction zone at any point in the time interval *T*, thus accounting for all the trajectories in Eq. (19) that contribute to association. In Fig. 3(a), we illustrate a single trajectory tracking the separation between a pair of particles versus time. When the particles are separated by less than *R*_max_, the probabilities differ for this trajectory to be sampled using the free and the exact propagator. To correct for this difference, we reweight the association probability.

**Fig. 4:**
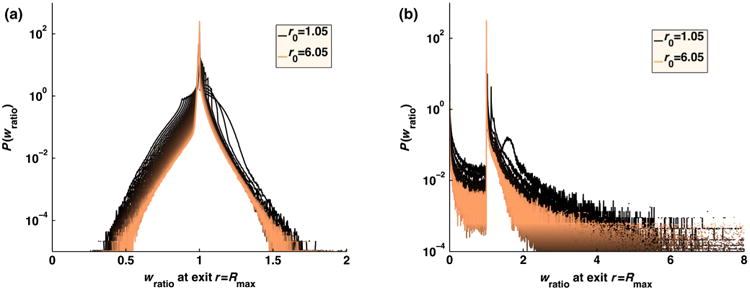
Distribution of exact to simulated trajectory probabilities on exiting the reaction zone for different initial separations *r*_0_, with each curve representing an increment of 0.1 nm in *r*_0_. These values reflect the degree of overlap between the exact and the simulated trajectories. If the reweighting-factor distributions are broad, the simulated trajectories will waste time on highly improbable trajectories, and miss out on some relevant trajectories, hence, undersampling in Eq. (19). (a) *k_a_* = 10.0 nm^3^/*μ*s, *D_AB_* = 20 nm^2^/*μ*s, Δ*t* = 0.1 *μ*s. (b) *k_a_* =1000.0 nm^3^/*μ*s, *D_AB_* = 1 nm^2^/*μ*s, Δ*t* = 0.01 *μ*s.

When trajectories exit the reaction zone, the value of their accumulated reweighting ratio will differ from one, but the exit ratio averaged over all trajectories will be one in the case of complete sampling. This average exit ratio, defined as $\langle P_{\text{exit}}(r_{0})\rangle =\int_{0}^{\infty }dt(p_{\text{irr}}(R_{\max},t|r_{0}))/(p_{\text{free}}^{*}(R_{\max},t|r_{0}))$, reflects the reweighting factors accumulated by trajectories that survived to the edge of the reaction zone *R*_max_. In Fig. 4(a), we show the distribution of these exit ratios for different initial starting positions *r*_0_. The average exit ratio is one because by construction the association probabilities of the reweighted free trajectories match those of trajectories created with the exact irreversible pair propagator. Therefore, the overall survival probabilities of the reweighted trajectories are exact. As a consequence, free trajectories with weights exceeding the probability of the exact trajectories are balanced by trajectories of low weight. The average ratio must be one to ensure the correct association probabilities.

As an important practical consequence of the average weight being one, all trajectory weights can simply be reset to one upon leaving the reaction zone. Consequently, their trajectory ratios are simply reinitialized to one once they reenter the reaction zone. As noted previously, the trajectory reweighting factor will be strictly equal to one for all sampling done outside the reaction zone where the free propagator correctly describes particle displacements.

#### Undersampling or nonoverlapping trajectories

**Fig. 5:**
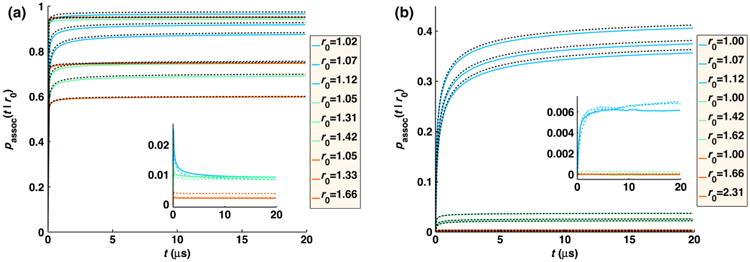
Time-dependent probability of association of particle pairs. (a) Same parameters as Fig. 1, but now with the time step Δ*t* = 0.001 *μ*s. For this smaller time step, the error increased, particularly for slower *D_AB_*. This is a result of incomplete sampling of the relevant trajectories and therefore incomplete reweighting of all trajectories. The error is larger for (a) *k_a_* = ∞, decreasing for the smaller association rate shown in (b) *k_a_* = 10 nm^3^/*μ*s. The insets show the difference between exact and simulated results.

It is possible that even trajectories limited to the reaction zone will be long enough that their probability of being sampled will not overlap with the exact trajectory probability distribution. In these circumstances, some of the sampled trajectories will not contribute to association [because the corresponding *p*_irr_(*r_T_*, *T*; *r*_*T*−Δ*t*_; *T* − Δ*t*;…; *r*_Δ*t*_, Δ*t*|*r*_0_) ≈ 0], which theoretically is fine, but other trajectories that do contribute to association will not be sampled [because $p_{\text{free}}^{*}(r_{T},T;r_{T-\Delta t},T-\Delta t;\ldots ;r_{\Delta t},\Delta t|r_{0})\approx 0$], which is problematic. As a result, the overall association rate [Eqs. (19) and (20)] will be slightly lower than Eq. (16). In 1D, this problem arises for all reactions, and therefore, trajectory reweighting would not be an accurate approach, although in 1D it is straightforward to use the exact propagator. In 3D, this issue emerges for systems with very short time steps, and slow diffusion, particularly when the association rate is high. In Fig. 4(b), we show how this lack of overlap in trajectories is manifested in the distribution of *P*_exit_. In Fig. 4(a), the simulations used a larger time step and the trajectory ratios are all distributed around one, with 〈*P*_exit_〉 = 1. For the short time step used in Fig. 4(b), the ratio becomes widely distributed, with the peak around zero indicating that several sampled trajectories have near zero probability according to the exact propagator. The value of 〈*P*_exit_〉 for these simulations is approximately 0.985, and therefore, some of the exact trajectories must not have been sampled. In Fig. 5, we find that the contribution to association that is lost from unsampled trajectories results in a small decrease in the total probability of association, which has a larger error than Fig. 1. The deviations of 〈*P*_exit_ (*r*_0_)〉 from one, where *P*_exit_ (*r*_0_) need only be evaluated for a single reactant pair, can thus be used as an indicator of trajectory sampling issues.

**Fig. 6:**
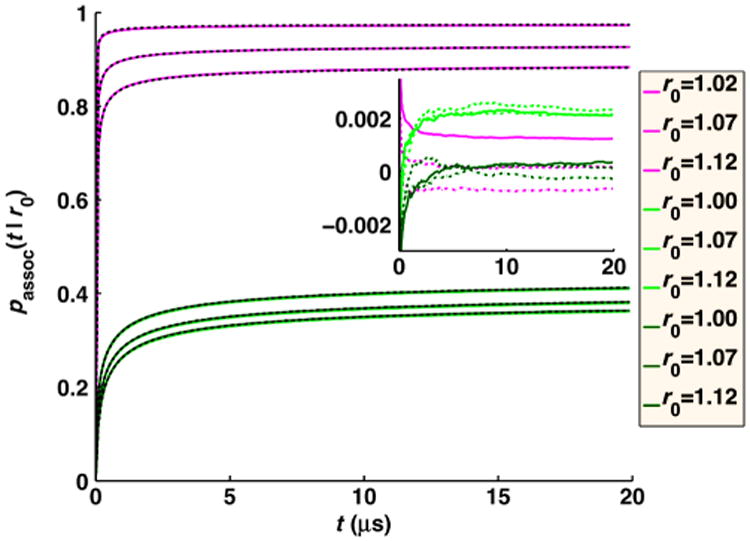
Time-dependent probability of association of particle pairs for the system of Fig. 5 with Δ*t* = 0.001 *μs* and *D_AB_* = 1 nm^2^/*μ*s, the parameter combination with the largest error, but now with the simulations adapted to reduce error. The RD simulations with absorbing BCs are in magenta and for *k_a_* = 10 nm^3^/*μ*s in light and dark green, with the exact solution shown as a black dashed line. To reduce the error for these conditions, we lowered the distance over which reweighting is applied. The magenta lines are simulations with the reweighting separation decreased from $3\sqrt{6D\Delta t}$ to $3\sqrt{6D\Delta t}$, but with the same reaction zone size. This means trajectories are reweighted for a shorter length of time. The light green lines are for *k_a_* = 10 nm^3^/μs and the reweighting separation decreased from $3\sqrt{6D\Delta t}$ to $3\sqrt{6D\Delta t}$. Another way to reduce error is to simply increase *k_a_*, and in dark green we show association for *k_a_* increased from 10 to 10.3 nm^3^/*μ*s. This approach is not applicable to absorbing BCs (*k_a_* cannot be increased) but works well for finite rates. The inset shows the difference between exact and simulated results.

We propose two ways to deal with the error arising from trajectory undersampling when small time steps are necessary and also diffusion constants are slow (*D*Δ*t* ∼ 0.001*σ*^2^). First, using the same size reaction zone *R*_max_, a smaller radial separation can be used inside which reweighting is applied, such that *R*_rewgt_ < *R*_max_. This will shorten the length of reweighted trajectories and can improve overall association rates, because fewer trajectories will be reweighted with low (approximately 0) ratios. In Fig. 6, we see that this approach improves agreement for simulations with *D* = 1 nm^2^/*μ*s, Δt = 0.001 *μ*s, and *k_a_* = ∞ and *k_a_* = 10 nm^3^/*μ*s, although not perfectly. Second, a larger intrinsic rate constant *k_a_* could be input to the program to bring the total association up to the desired level. In Fig. 6, the dark green lines report simulations with *k_a_* = 10.3 nm^3^/*μ*s and very small error. However, the choice of reweighting zone radius *R*_rewgt_, or increased association rates, was only determined through trial and error.

### C. Including pairwise interaction potentials

A powerful aspect of the FPR method derived above is its general applicability to propagators beyond just the particular GFs defined in Eqs. (7) and (11). To demonstrate this, we consider the introduction of a radially symmetric interaction potential *V*(*r*) between each particle pair. The pair diffusion equation is then given by

(21) $$\frac{\partial p(r,t|r_{0})}{\partial t}=(\frac{\partial }{\partial r}+\frac{2}{r})D(\frac{\partial }{\partial r}+\beta \frac{\partial V(r)}{\partial r})p(r,t|r_{0}).$$

The reaction between the particles, again defined by the flux across the contact surface, gives rise to the radiation boundary condition of

(22) $$4\pi \sigma^{2}D(\frac{\partial }{\partial r}+\beta \frac{\partial V(r)}{\partial r})p(r,t|r_{0}){|}_{r=\sigma }=k_{a}p(\sigma ,t|r_{0}).$$

This PDE is not generally solvable analytically for the GF or the survival probabilities, and therefore, we do not have comparable closed-form solutions corresponding to Eqs. (7) and (9). We instead solve for these functions numerically using the method of Zhou [31].

To propagate our particles in the simplest manner possible, we again use the free-diffusion propagator but with a drift term added, such that the update scheme for the particles consists of the moves

(23) $$r(t+\Delta t)=r(t)+D\beta F(r(t))\Delta t+\sqrt{2D\Delta t}R,$$

where **F**(**r**) = −*dV*(**r**)/*d***r** is the force vector and ***R*** is a vector of normally distributed uncorrelated random numbers with mean zero and unit variance in each dimension. This update scheme samples from the distribution given by

(24) $$p_{\text{free}}^{Vr}(r,\Delta t|r_{0})=\frac{1}{4\pi r[r_{0}+D\Delta t\beta F(r_{0})]\sqrt{D}}\frac{1}{\sqrt{4\pi t}}\times [\exp(-\frac{{\left[r-r_{0}-D\Delta t\beta F(r_{0})\right]}^{2}}{4Dt})-\exp(-\frac{{\left[r+r_{0}+D\Delta t\beta F(r_{0})\right]}^{2}}{4Dt})]$$

for a single time step Δ*t*, where *F*(*r*_0_) = −*dV*(*r*_0_)/*dr*_0_. We note that while the BD update scheme in Eq. (23) will sample from this distribution for a single step, this distribution is not generally accurate for the particle positions after multiple steps if *F*(*r*) is not constant over the length of the displacement. Whereas this does not affect the FPR theory, variation in *F*(*r*) over the displacement does place a further limitation on the size of the time step, and for long-range forces may affect the limiting size of the reaction zone, which we discuss below.

**Fig. 7:**
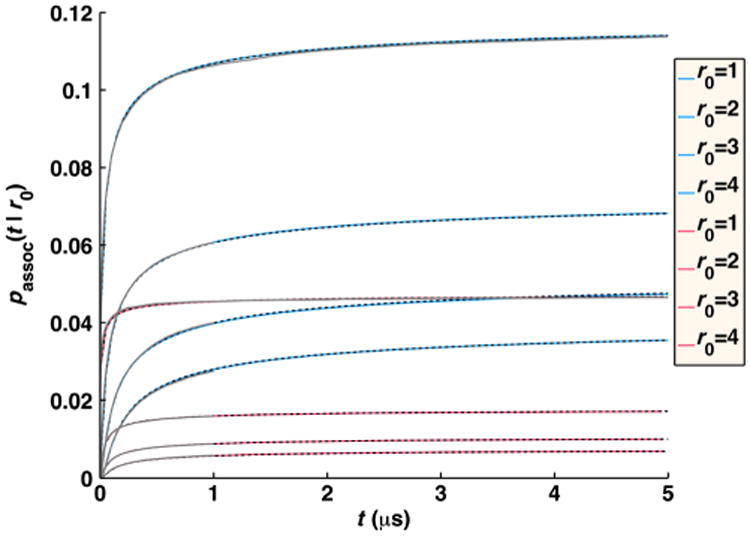
Probability of two particles with an additional Coulomb interaction, *βV*(*r*) = *r_c_/r*, having associated between times 0 and *t*, given they were separated by a distance *r*_0_ at *t* = 0. Blue lines are for an attractive interaction with *r_c_* = −1, and pink lines are for a repulsive interaction with *r_c_* = +1. The binding radius is *σ* = 1 nm, *D_AB_* = 50 nm^2^/*μ*s, *k_a_* = 50 nm^3^/*μ*s, and Δ*t* = 0.01 *μ*s. The black dashed lines represent simulations propagated using the “exact” numerically solved GF to update the particle separations. The extremely close agreement between the reweighted probabilities and the exact GF updates demonstrates that trajectory overlap is not an issue for this time step. These simulations are averaged over 10^7^ trajectories, each initialized to the prescribed *r*_0_. The gray lines represent the association probability calculated using the BD scheme of Zhou [31]. These BD simulations are much slower than the FPR simulations due to the very short time step necessary, and for *r*_0_ > 1 are propagated out to only *t* = 1 *μs* for 3 × 10^5^ trajectories. For *r*_0_ = 1, we collected 10^5^ trajectories out to 10 *μ*s.

The size of the reaction zone, whether or not a potential is present, needs to be large enough to account for the effects of the reactive barrier at *r* = *σ*. Outside of the reaction zone, the particles will have essentially zero probability of reaching the reactive barrier (by construction) and, therefore, their dynamics will correctly be described by the solution to the diffusion equation without any reactive BC. For the case of no potential or a shorter-range or truncated potential that does not extend beyond the reaction zone, this solution is given by the free-diffusion propagator in Eq. (5). However, if the potential is long range, such that it acts even beyond the reaction zone separation, there are two additional considerations. First, if the force at these longer ranges still varies significantly over the length of the diffusional displacement, then sampling from the free propagator with drift [Eq. (24)] may not agree with the true irreversible propagator, and should therefore be reweighted. This could be accounted for by extending the reaction zone until the two distributions match and/or shortening the time step. Second, and more critically, accounting for these long-range forces means one must measure the effects of pairwise forces well beyond the reaction zone. This presents no issues for a single particle pair, and in Fig. 7, we show that trajectory reweighting works just as well in the presence of either a repulsive or an attractive Coulomb potential.

### D. Time step limitations

For a single pair of particles, the time step for the FPR method formally has no inherent limits. In practice, the possibility of undersampling reactive trajectories means some time steps could give rise to decreased association rates. As noted above, trajectory overlap issues can arise in systems without interaction potentials when the time step is very small and long trajectories build up in the reaction zone. When an interaction potential is present, on the other hand, a large time step may result in significant discrepancies between the single-step propagators if the force varies widely over the course of a single displacement. Hence when a potential is present, extra care needs to be taken to ensure the time step is not so large that overlap becomes a problem. For the Coulomb potential modeled in Fig. 7, a time step of 0.01 *μ*s was small enough to avoid these issues, which could be anticipated from the sufficient similarity between the single-step propagators.

For many-particle systems, the time step for propagating the spatial dynamics of the system will ideally be chosen as large as possible in order to rapidly reach relevant time scales on the order of seconds, minutes, or longer, while still faithfully modeling the evolution of the system through this interval. The time step should not be larger than the time scale of the fastest process in the system, since otherwise the assumption of at most one association or dissociation event per particle per step will be violated. This tends to be an upper bound that typically exceeds the more limiting constraint of ensuring that only a single pair of particles is close enough to react in a single time step. This latter requirement is necessary because we need to break up the system into pairwise interactions in order to use the equations of Sec. II A–II C. Therefore, the time step should be chosen to reduce particles having multiple reactive partners in a single time step, which will depend on the particle number densities *ρ_A_* and diffusion coefficients *D_A_*. Assuming uniform spatial distributions of all particles, the number of reactive partners of any type in a thin spherical shell of thickness *∊* around a particle of type *A* is approximately $N_{A,\text{rct}}\approx \sum_{B}4\pi \sigma_{AB}^{2}∊\rho_{B}$, where the sum extends over all particle types *B* reacting with *A*, and *σ_AB_* is the binding radius for the reactant pair. The shell thickness *∊* should encompass the full reaction zone *R_max_*. A reasonable estimate of *R*_max_ is to choose a multiple of the average displacement, $\sqrt{6D_{AB}\Delta t}$, with *D_AB_* the combined diffusion constant. The value $R_{\max}=\sigma_{AB}+3\sqrt{6D_{AB}\Delta t}$ [30] is sufficiently large that particles outside this separation will be very unlikely to collide in a single step. According to this definition of $R_{\max},∊=3\sqrt{6D_{AB}\Delta t}$, and we have $N_{A,\text{rct}}\approx \sum_{B}4\pi \sigma_{AB}^{2}(3\sqrt{6D_{AB}\Delta t})\rho_{B}$. Many-body effects are minimal if, for all particles, this number is less than one, requiring that the time step satisfies $\Delta t<\min_{A}{\left(\sum_{B}12\pi \sigma_{AB}^{2}\sqrt{6D_{AB}}\rho_{B}\right)}^{-2}$, such that, on average, each particle has at most a single reactive partner.

## III. Implementation of FPR Dynamics

### A. Implementation for one particle pair

#### Association and reweighting

When two particles are separated by a distance less than *R*_max_, where *R*_max_ defines the reaction zone, their probability of association is calculated and reweighted. The size of *R*_max_ is chosen to encompass the range over which the particles could diffuse to encounter one another, and, therefore, where the association probability is nonzero. We generally use $R_{\max}=\sigma_{AB}+3\sqrt{6D_{AB}\Delta t}$ [30], as it represents a good estimate of where the association probability drops to zero and is a simple function of the known reaction parameters. A larger value of *R*_max_ does not change the behavior of the reweighting method, although it does mean particles have to be treated as pairs rather than free particles at larger separations, which results in a slightly slower position update. Two particles starting from outside their reaction zone have close to zero probability of encountering one another within one time step, and their displacements are thus accurately described by free diffusion. When a pair of particles first moves close enough for association (*r_AB_* < *R*_max_), their reweighting ratio is initialized to one (*w*_ratio_ = 1), as they were previously undergoing free diffusion. Whenever the pair associates, the “complex” is placed between the two particles at the point defined by each particle's diffusion constant fraction *D_A_/D_AB._* If the pair does not associate, their current separation is stored, as is the probability that they survived (i.e., did not associate) within this time step. In the next step, if the pair remains in the reaction zone, their association probability is first calculated using Eq. (9). This value is then reweighted by $w_{\text{ratio}}=p_{\text{irr}}(r_{t}|r_{t-\Delta t})/p_{\text{free}}^{*}(r_{t}|r_{t-\Delta t})$, where $p_{\text{free}}^{*}(r_{t}|r_{t-\Delta t})$ normalizes to the survival probability from the previous position and *r_t–_*_Δ_*_t_* is the separation stored from the previous time step. If the pair again does not associate, their current separation is stored, as is the value of the trajectory ratio *w*_ratio_ and the probability they survived this step, which is given by *S*(Δ*t*|*r_t_*) = 1 – *w*_ratio_*p*_assoc_(Δ*t*|*r_t_*). In the next step, the same procedure applies, except the value of the trajectory ratio for the current step is multiplied by the ratio from all the previous steps, $w_{\text{ratio}}=p_{\text{irr}}(r_{t}|r_{t-\Delta t})/p_{\text{free}}^{*}(r_{t}|r_{t-\Delta t})*p_{\text{irr}}(r_{t-\Delta t}|r_{t-2\Delta t})/p_{\text{free}}^{*}(r_{t-\Delta t}|r_{t-2\Delta t})$. In this way, the probability of both the full exact and free trajectories is accumulated in the reweighting ratio, where, thanks to the Markov property, only the probability of the current step having propagated from the immediately preceding step needs to be calculated. When the particles separate farther than *R*_max,_ their trajectory ratio is reset to one, the average value at exit.

#### Position propagation

All position updates for particles of a given type *A* are determined by independent Gaussian random numbers with a mean of zero and a standard deviation of $\sqrt{2D_{A}\Delta t}$ in each dimension. If two particles overlap after an attempted position update (*r_AB_* < *σ_AB_*), the positions are rejected and new updates are chosen for both particles from their previous positions. The reason both particle positions must be updated is because the separation and overlap between the pair depends on *D_AB_* and not just *D_A_* around a fixed object.

#### Dissociation

Once a pair of particles is bound, their dissociation is modeled as a Poisson process with the rate given by the intrinsic dissociation rate *k_b_* (it does not account for diffusion of particles away from one another). At each step after a pair associates, particles attempt to dissociate with probability given by *p*_diss_(Δ*t*) = 1 – exp(*–k_b_*Δ*t*). When they do dissociate, they are placed at the contact separation of *σ*. The dissociated particles are displaced relative to the complex by selecting a vector uniformly on the sphere of radius *σ* and moving them away from the complex along this vector by each particle's diffusion constant fraction *D_A_*=*D_AB_*. To ensure detailed balance, dissociation and association processes are inverses of each other. Because they dissociate in the time interval for this step, they do not attempt to reassociate until the next step.

The process can be described by the following steps given the pair started inside or outside the reaction zone.

Case A: Particles are separated by a distance larger than *R*_max_.

(1) Each particle is propagated according to the free propagator. If a force is present, a drift term is included in the position update.

Case B1: Particles are separated by a distance less than *R*_max_, but previously were outside of this reaction zone.

(1) The association probability for the particles are calculated. (2) This pair's reweighting ratio is initialized to one. This ratio, their current separation, and their association probability are stored. (3) If *p*_assoc_ > URN, then (a) particles are moved along their separation vector to form a new complex; (b) else, new positions are sampled for each particle from the free propagator. (4) If particles did not associate and the positions of the pair overlap (*r_AB_ <σ_AB_*), both position updates are rejected and new positions are selected until there is no overlap.

Case B2: Particles are separated by a distance less than *R*_max_, and previously were inside the reaction zone.

(1) The current ratio of the particle pair given their current separation and their stored previous separation is calculated. (2) The new trajectory reweighting ratio *w*_ratio_ is defined by the old stored value times the current ratio. (3) The association probability for the particles is calculated and scaled by the new trajectory reweighting ratio *w*_ratio_. (4) This pair's new reweighting ratio, their current separation, and their scaled association probability are stored. (5) If *w*_ratio_ * *p*_assoc_ > URN, (a) particles are moved along their separation vector to form a new complex; (b) else, new positions are sampled for each particle from the free propagator. (6) If particles did not associate and the positions of the pair overlap (*r_AB_ <σ_AB_*), both position updates are rejected and new positions are selected until there is no overlap.

Case C: Particles are bound in complex.

(1) If 1 – exp(*–k_b_*Δ*t*) > URN, particles dissociate. New positions for each particle are selected uniformly around the central bound complex to a final separation *σ_AB_*. (2) Else, a new position is sampled for the complex from the free propagator.

URN is a uniform random number in the interval [0, 1).

### B. Implementation of FPR for multiple particles

Here, we propagate systems of many particles with a fixed time step. Initially, all particles are distributed so that no reactive pairs overlap with one another. At each time step, each particle can undergo one reactive event, either association or dissociation, and otherwise it diffuses. For each time step, first, all particles that are bound are tested for dissociation. Second, all separations between pairs of interacting particles are measured. When there are no long-range interactions, this calculation can be sped up, e.g., by breaking the simulation volume into spatial cells and only testing pairs in the same and neighboring spatial cells. If the distance between a pair of interacting particles is less than *R*_max_ for that interacting pair, the probability of the pair associating is calculated, reweighted, and stored. The partner list is also stored for the final check of overlapping positions after diffusion. If a particle is not within *R*_max_ of any of its interaction partners, it is freely diffused. Third, we loop over all particles, and if they have crossed within the reaction zone of any interaction partners, the probabilities of associating with each potential partner are calculated. Ideally, each particle would have at most a single partner in its reaction zone, such that the reaction is truly defined by the equations in Sec. II. While the time step should be chosen to best ensure only such pairs of particles, density fluctuations can result in a particle having multiple reaction partners in its reaction zone. We choose to deal with multiple partners by summing over each of their individual association probabilities, and the total association probability is compared to a URN. If the URN is less than the total association probability, the reaction partner is chosen based on the probability interval encompassing the URN. The associating particle pairs are moved to a position between them and their reaction probability is subtracted from the lists of all other potential interaction partners. If the URN is greater than the association probability, no association occurs and the particle's position is updated via the free propagator. Fourth, once all particles have attempted association, the new position of all particles must be tested for overlap. To do this, we again loop over all particles, and over all interacting partners stored, as within the reaction zone. If a particle *A* has overlap with particle *B*, both their positions are rejected and new ones are sampled, and overlap is again checked against all of *A*'s partners. However, once a particle has moved to a position without overlap, we do not allow it to move again within that time step. When it becomes *B*'s turn to check overlap, *B* still avoids the position of *A*, but since *A* has already moved, at this point only *B* can move. If *B* does not overlap any partners, it remains at the position sampled during *A*'s turn. This is necessary to ensure that all particles avoid overlap with all partners but still only make a single displacement. If each particle has only a single partner in its reaction zone, this procedure will reproduce the simple pairwise scheme outlined in the previous section.

#### Considerations for many particle systems

The FPR method solves the full many-body problem by breaking it into a series of two-body problems. As noted above, whenever a particle *A* has more than one reactive partner to attempt association with, the pairwise assumption is violated because the problem is no longer two body. In the FPR method, we assume that the probabilities of trajectories leading to reaction with each partner can be summed, but this might not be strictly true, as the trajectories may overlap one another. Also, sampling a new position for the *A* particle will require avoiding both reaction partners, which means the position may deviate from that distribution defined by the pair propagator. To minimize these problems, the time step should be limited by the considerations described in Sec. II D. The excellent agreement between our many-body simulations below and known theoretical results suggests that the effects of these non-two-body encounters is marginal.

In the FPR solution to the full many-body problem, the distribution of particles is not exact in space, because at small separations between particles the free propagator is used to update positions rather than the exact two-body propagator. However, outside of these short separations, the particles do propagate correctly and, as we see in Sec. IV, both equilibrium and relaxation properties of the system that result from diffusion are well reproduced by the FPR method.

### C. Implementation of FPR for multiple particles with a long-range potential

Within the reaction zone, FPR will produce the correct association rates for particle pairs even when an interaction potential is present. Outside of the reaction zone, if a long-range potential still operates, these long-range forces must somehow be accounted for. For a single pair of particles, this can be correctly captured by adding a drift term to the free diffusion of each particle when outside the reaction zone via Eq. (23). For many particles, however, this long-range interaction disrupts the assumption that the system can be broken into isolated pairs of interacting particles and will require approximate treatments for all methods (including GFRD) that rely on pairwise interactions. One approximate strategy is to simply extend the two-body method by summing up all the forces a particle feels from all other reaction partners in the system, regardless of whether they are in the reaction zone. The reactions between particles are still treated in the exact same way as Secs. III A and III B, but the update to each particle position will include a drift term that has a total force, rather than the force from the single reaction partner,

(25) $$r_{i}(t+\Delta t)=r_{i}(t)+D\beta \sum_{j=1,j\neq i}^{N}F(r_{ij}(t))\Delta t+\sqrt{2D\Delta tR}.$$

Each reactive pair now interacts within the field generated by all the long-range forces. For large *N* systems, the radial symmetry should give rise to an average force of zero, such that, effectively, only the partner within the reaction zone will contribute to the force. Outside of the reaction zone, the drift term may become quite small relative to the diffusional displacement, but because it is directional rather than random, it cannot be wholly neglected.

An alternate strategy, useful, in particular, for shorter-ranged interactions and more in keeping with the overall reweighting approach, is to maintain the same behavior as in Sec. IIIB, but attempt to correct for the use of the free propagator when particles are outside the reaction zone. Specifically, when particles enter the reaction zone, they have a trajectory ratio given by $[p_{\text{free}}^{\text{Vr}}(R_{\max},t|r_{0}>R_{\max})/p_{\text{free}}(R_{\max},t|r_{0}>R_{\max})]\neq 1$. Because reweighting is not applied outside of the reaction zone, this results in having an incorrect accounting of the density of particles entering the reaction zone. We therefore attempt to correct for this error by initializing the reweighting ratio for particles entering the reaction zone via the density ratio, rather than initializing to one. Using the equilibrium probability distribution of particles at the entrance to the reaction zone, this ratio is given by

(26) $$w_{\text{ratio}}=\exp[-\beta V(R_{\max})].$$

This approach allows us to use the significantly faster short-range pairwise calculation described in Sec. III B, because only the forces of a particle in the reaction zone need be calculated. In this approach, we note that although this will improve the association rate between particles, the density distribution of particles will be off at all distances since the long-range forces are ignored beyond the reaction zone. Using the summation method above should better reproduce the actual long-range particle density.

### D. Simulation setups

#### Pseudo-first-order reactions

For simulations of pseudo-first-order reactions, a single *A* particle is centered in a box with a uniformly distributed number of *B* particles placed randomly inside the box and outside of the volume $\frac{4}{3}\pi \sigma^{3}$ of the *A* particle. Because the *B* particles do not interact with one another, they do not exclude other *B* particles. The *A* particle starts as unbound. We set the reaction zone to a separation of $R_{\max}=\sigma_{AB}+3\sqrt{6D_{AB}\Delta t}$. Inside the reaction zone, the *B* particles attempt to associate with the *A* particle, and are otherwise moving diffusively, avoiding overlap with *A*. When *A* is bound, the free *B* particles continue to diffuse and avoid overlapping the bound *A* molecule. Following Popov and Agmon [25], the box size is chosen to ensure that the particles at the edge of the box will not diffuse all the way to the center during the duration of the simulation *T*_max_, with box sizes of approximately $2\sqrt{6D_{AB}T_{\max}}$, and larger boxes tested for some systems as well. We then collected the change in *A*'s bound and free status versus time for approximately 300 000 trajectories, with newly initialized positions for each trajectory.

#### Reversible simulations

The algorithm was propagated as described above in methods for multiparticle systems. A time step of 0.1 *μ*s was used unless noted with simulations of 2000 or 100 000 *A* and *B* particles. For some cases, the particles *A* and *B* also avoided overlap with the product *C*, because if *C* dissociated and was not avoiding *A* and *B*, the products might overlap a reactant. For dilute systems, this is not a major issue. To minimize the slow-down of the simulations, particle pairs were simply pushed to contact before attempting to associate.

#### Interaction potential simulations

The simulations were initialized as above, with the long-range forces treated in the two ways discussed in Sec. III C. The reaction zone could still be defined as $R_{\max}=\sigma_{AB}+3\sqrt{6D_{AB}\Delta t}$ to correctly encompass the collision length of the particles but was also tested at $R_{\max}=\sigma_{AB}+4\sqrt{6D_{AB}\Delta t}$. The time step was short enough that this separation would still prevent most many-body interactions. The results were not sensitive to this extension of the reaction zone.

### E. Numerical GF calculations

When particles exert forces onto each other while diffusing and reacting at contact, the GF can be solved numerically using the method of Zhou [31]. For the fixed time step used in FPR, we collect both the survival probability determining the reweighting factor and the GF as a function of initial particle distances *r*_0_ in the reaction zone, ranging from *σ* to *R*_max_ in increments of Δ*r*_0_ = 0.05 nm. The method requires propagating a single pair of particles using BD updates [Eq. (24)] with a very small time step, while enforcing the reactive boundary in a small shell around *r* = *σ*. For details, we refer the reader to the original Ref. [31]. This method improves in accuracy as the size of the shell approaches zero, although this requires smaller time steps. The size of the shell *ε* is chosen as *∊* = (*fD*4*πσ*^2^/*k_a_*), where *f* is some factor ≪1. We use *f* = 0.001, and the time step for the BD updates is defined by Δ*t* = *f∊*^2^/(2*D*). A smaller value of *f* = 0.0005 gives indistinguishable results. This process must be repeated for each *r*_0_ value multiple times to smooth out the GF, and we repeated each pair approximately 600 000 times. Each trajectory is simulated either until association occurs or the time reaches the target time step for the FPR simulations, which is Δt = 0.01 *μ*s. For the value of δ*t* noted above, the longest trajectories are ∼6 × 10^6^ steps. The survival probability is calculated as the number of trajectories that survive to Δ*t* given *r*_0_, and the GF is the distribution of final particle positions that survive to Δ*t* given *r*_0_.

These distributions can then be used in our FPR method by storing them in look-up tables. To smooth them out and extrapolate the distributions to zero, we instead fit both the survival probability and the GFs. The fits are not meant to provide physical insight into the behavior, but specifically to provide smooth representations of the numerical data. The survival probability is fit to Eq. (9), with *k_a_* and *D* treated as fit parameters. The GFs are fit either to Eq. (7) multiplied by a scale factor or to Eq. (24) multiplied by a scale factor. Hence, each GF fit has three flexible parameters. By separately fitting over the first and last halves of the distributions, the fits are excellent.

## IV. Results

As shown in the following, numerical simulations with the FPR algorithm agree very well with available theoretical results over a broad range of particle solutions and reaction types. We are able to reproduce both equilibrium and time-dependent relaxation properties of dense solutions of particles. The largest deviations occur in extremely dense systems (concentrations in the high mM range) with slow diffusion (on the order of 10^−8^ cm^2^/s). Such high concentrations may occur when considering all the components of the cytosol, but when considering subsets of reactive species, individual protein concentrations tend to be much lower and diffusion constants in solution tend to be significantly higher. Furthermore, the deviations for these systems are relatively small and result in systematically decreased association, as discussed in Sec. II B. Therefore, the desired association rate can be achieved by using a slightly higher effective value of *k_a_* for dense and slow systems. Alternatively, a smaller radius *R*_rewgt_ for applying reweighting can be used to reduce the error.

### A. Irreversible reactions A + A → 0

For a solution of A particles that diffuse and react with one another to form a nonreactive product (annihilation), the relaxation of the many-body system is well described by Smoluchowski theory at all time scales [6,7,32]. This theory defines the time-dependent rate *k*(*t*) of association to a central particle in a solution of initially uniformly distributed particles. The rate derives from the solution to Eq. (2) for the density distribution of particles *ρ*(*r*, *t*) with the initial condition *ρ*(*r*, 0) = 1 and the BC *ρ*(*r* →∞,*t*) = 1 [33]. The time-dependent rate *k*(*t*) is the flux of density at the binding radius *σ* and can then be used to describe the change in particle concentration *A*(*t*) over time via the ordinary differential equation

(27) $$\frac{\text{dA}}{\text{dt}}=-k(t)A{\left(t\right)}^{2}.$$

As the distribution of particles evens out, the rate approaches a time-independent steady-state value that is used in chemical kinetics.

The rate of association depends on whether the particles always react on contact (absorbing BC), or only react with the intrinsic rate constant *k_a_* (radiation BC). For absorbing boundary conditions, the time-dependent rate is given by

(28) $$k(t)=4\pi \sigma D(1+\frac{\sigma }{\sqrt{\pi Dt}}),$$

and has a steady-state value of *k_D_* = 4*πσD*, with *σ* = *σ_AA_* and *D* = *D_AA_*.

For radiation boundary conditions with an intrinsic rate constant *k_a_*, the time-dependent rate is given by

(29) $$k(t)=k_{D}(\frac{k_{a}}{k_{a}+k_{D}})(1+\frac{k_{a}}{k_{D}}\exp(\lambda t)\text{erfc}(\sqrt{\lambda t})),$$

where *λ* = (*D/σ^2^*)(1 + (*k_a_/k_D_*))*^2^* and the steady-state value *k*_on_ = ((1/*k_a_*) + (1/*k_D_*))^−1^ reflects the time to react due to both diffusion and reactivity at contact [33].

**Fig. 8:**
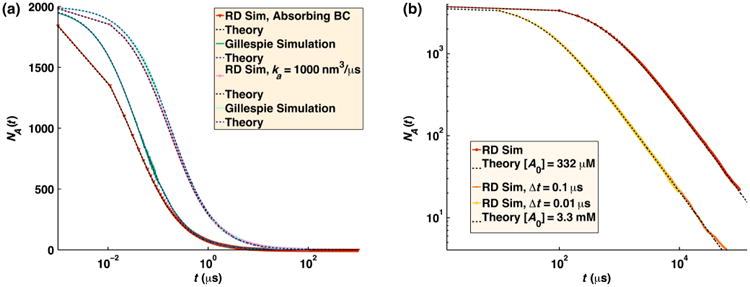
Irreversible reaction *A* + *A* → 0. *A*(*t*) versus time for (a) absorbing BC, Δ*t* = 0.001 *μ*s and *D_AB_* = 200 nm^2^/*μ*s. RD simulations are shown in red for a 16.6 mM concentration of *A* particles. The solution to Eq. (27), $A(t)={\left\{(1/A_{0})+k_{D}[(t+2\sigma \sqrt{t}/\sqrt{D_{\text{tot}}\pi })]\right\}}^{-1}$, is plotted in black dashed lines. In pink, we show RD simulations for *k_a_* = 1000 nm^3^/*μ*s, Δ*t* = 0.001 *μ*s, and *D_AB_* = 100 nm^2^/*μ*s, and the theoretical solution to Eq. (27) given by $A(t)={\left\{(1/A_{0})-k_{D}[k_{a}/(k_{a}+k_{D})](k_{a}/k_{D}\lambda )+k_{D}[k_{a}/(k_{a}+k_{D})][t+(k_{a}2\sqrt{t}/k_{D}\sqrt{\lambda \pi })+(k_{a}/k_{D}\lambda )\exp(\lambda t)\text{erfc}(\sqrt{\lambda t})]\right\}}^{-1}$ in dashed black. For comparison, we show results from Gillespie simulations for simple chemical kinetics (cyan, no symbols) of both reactions and the corresponding solution to Eq. (27) with the steady-state rate in blue dashed lines. (b) RD simulations with *k_a_* = 10 nm^3^/*μ*s, Δ*t* = 0.1*μ*s, Δ*t* = 0.01 *μ*s, and *D_AB_* = 20 nm^2^/*μ*s demonstrating the long-time steady-state decay at 332 *μ*M concentration of *A* particles (red circles) and 3.3 mM (orange and yellow data simulated with different time steps).

In Fig. 8, we compare numerical simulations using FPR to the theoretical results for *A*(*t*) and find extremely good agreement. To highlight the contribution of diffusion to the relaxation process, we also show the relaxation from chemical kinetics simulations, where the steady-state rate *k*(*t →* ∞) is used in the solution to Eq. (27). The effect of diffusion on the relaxation process is most pronounced with absorbing boundaries, but is also visible for radiation boundaries [Fig. 8(a)]. At longer times, and for systems with slower reaction rates and faster diffusion, the processes converge to the steady-state relaxation of chemical kinetics [Fig. 8(b)].

### B. Reversible reactions *A* + *B* ⇄ *C*

In the case of solutions of particles that can both associate and dissociate, the analytical description of the full time-dependent relaxation of the system to its well-defined equilibrium concentrations is complicated by the reappearance of particles after dissociation. However, in the case of pseudo-first-order reactions, where one particle is in great excess over another (B ≪ A) such that its concentration is essentially unchanged as the reaction evolves, theoretical results have been derived [8,34].

Before we compare the time-dependent relaxation, we first simply test whether proper equilibrium is reached for a simple target problem with a central particle *A* surrounded by a uniform concentration of *B* particles (noninteracting with one another) at various reaction rates, diffusion constants, and time steps. The equilibrium probability of a single particle *A* being bound when surrounded by a uniform distribution of *B* particles at concentration *B*_0_ is given by

(30) $$p_{\text{bound}}(B_{0})=1-A_{\text{eq}}=\frac{K_{\text{eq}}B_{0}}{\left(1+K_{\text{eq}}B_{0}\right)}.$$

**Fig. 9:**
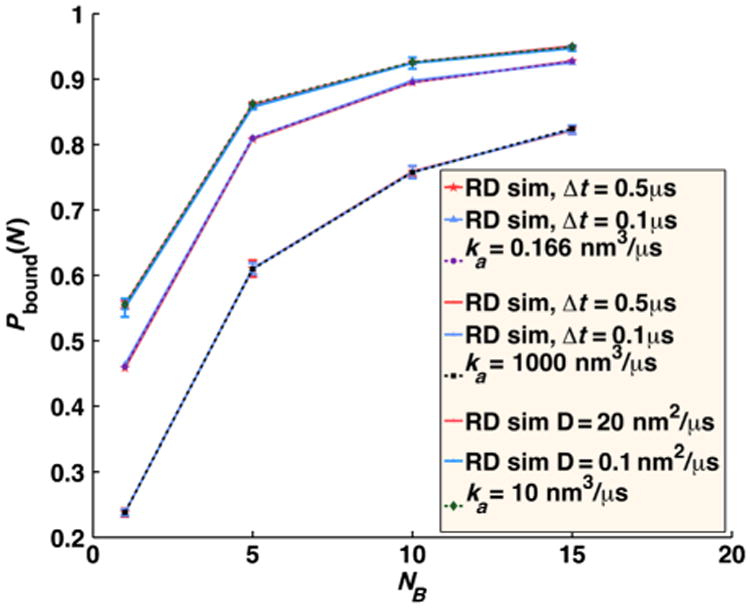
Equilibrium association probability [Eq. (24)] for systems with a central *A* particle and *N_B_* = 1, 5, 10, and 15 *B* particles uniformly distributed throughout the volume. Lines are guides to the eye. Dashed lines connect the theoretical values, and red and blue lines connect the simulated values shown with error bars. From top to bottom, the green diamonds are results for *k_a_* = 10 nm^3^/*μ*s us, *k_b_* = 1 s^−1^, and *V* = 8 × 10^6^ nm^3^, and the corresponding RD simulations use Δ*t* = 0.1 *μs* and *D_AB_* = 20 and 0.1 nm^2^/*μ*s is. The purple circles are *k_a_* = 0.166 nm^3^/*μ*s, *k_b_* = 0.1 s^−1^, and *V* = 1.95 × 10^6^ nm^3^, and the corresponding RD simulations use *D_AB_* = 5 nm^2^/*μ*s, Δ*t* = 0.5 *μ*s, and Δ*t* = 0.1 *μ*s. The black squares are *k_a_* = 1000.0 nm^3^/μs, *k_b_* = 50 s^−1^, and *V* = 64 × 10^6^ nm^3^, and the corresponding RD simulations use *D_AB_* = 20 nm^2^/*μ*s, Δ*t* = 0.5 *μ*s, and Δ*t* = 0.1 *μ*s. The binding radius is always 1 nm. Simulations are run for the duration of 5000 binding events to converge the bound probability, and 10–20 different simulations were run for each data point.

In Fig. 9, we compare this quantity with FPR numerical simulations for dilute concentrations of *B* particles. The agreement is excellent, for fast and slow reaction rates, and different time steps.

**Fig. 10:**
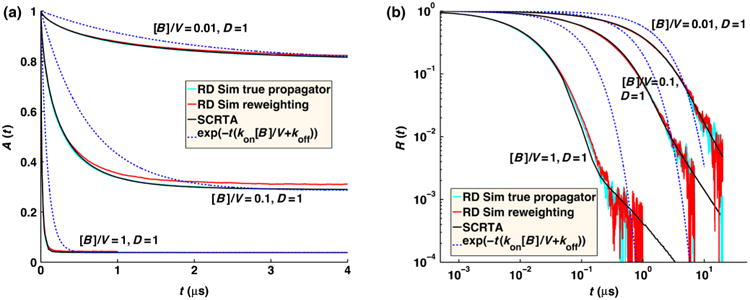
Pseudo-first-order reactions with a central *A* particle surrounded by a uniform distribution of *B* particles at densities *B*_0_/*V* nm^−3^. (a) Concentration *A*(*t*) of free *A* particles versus time, approaching the equilibrium value of *A*_eq_ = 1/(1 + *K*_eq_*B*_0_/*V*). RD simulations are in red, and show some error, with *A*_eq_ being too high as association is too low. Cyan are simulation with displacements sampled from the true propagator, black is the SCRTA solution [8], and dashed blue is the relaxation obeying chemical kinetics. (b) Relaxation *R*(*t*) showing the approach to equilibrium.

In Fig. 10, we plot the concentration *A*(*t*) of free *A* particles and the full time-dependent relaxation to equilibrium *R*(*t*) = [(*A*(*t*) *– A*_eq_)/(*A*(0) – *A*_eq_)], for the pseudo-first-order target problem (*D_A_* = 0). We compare numerical FPR simulations to the analytical self-consistent relaxation time approximation (SCRTA) due to Gopich and Szabo [8]. This theory was shown to quite accurately agree with previous numerical simulations of even very dense systems of *B* particles [25]. In addition to simulating with the FPR algorithm, we also run simulations that sample particle separations from the exact radially symmetric GF [Eq. (7)], as was done similarly in previous numerical simulations [25]. We note that this approach is relatively simple only for this target problem when the angular degrees of freedom (DOF) can be ignored and, hence, only the radial DOF need be sampled, when interactions between *B* particles are ignored, and when only a single *A* molecule interacts per time step. For more heterogeneous systems, if one wishes to sample from the exact GF, an approach such as GFRD [30] must be used.

We find that for the small time step necessary for these very dense systems (a 1 ns time step is used for the solutions of 0.167*M* to 1.7*M* concentrations) with slow relative diffusion (*D* = 1 nm^2^/*μ*s), the equilibrium values of free *A* are too high, as association is undersampled [Fig. 9(a)]. Because *R*(*t*) should go to zero at equilibrium, with our simulated relaxation data, we subtract off the simulated *A*_eq_ obtained at long times, rather than the exact value given by Eq. (30). We show in Fig. 10(b) that the relaxation for these very dense systems is in close agreement with the simulations using the true propagator and with the SCRTA solution. The long-time asymptotics correctly display the power-law relaxation to equilibrium that occurs in diffusive systems. For comparison, we also plot the relaxation for homogeneous chemical kinetics given by *R*(*t*) = exp[–*t*(*k*_on_*B*_0_ + *k*_off_)], where *k*_on_=(1/*k_a_*)+(1/*k_D_*)^−1^, *k*_off_ ((1/*k_a_*)+(*K_eq_*/*k_D_*))^−1^, and *K_eq_* = (*k_a_*/*k_b_*) = (*k_on_/k_o_*_ff_). At these solution conditions, the reaction diffusion (RD) simulations collected using our FPR algorithm show clear differences from chemical kinetics, with an initial rapid relaxation followed by the slower power-law relaxation just before equilibrium is reached.

**Fig. 11:**
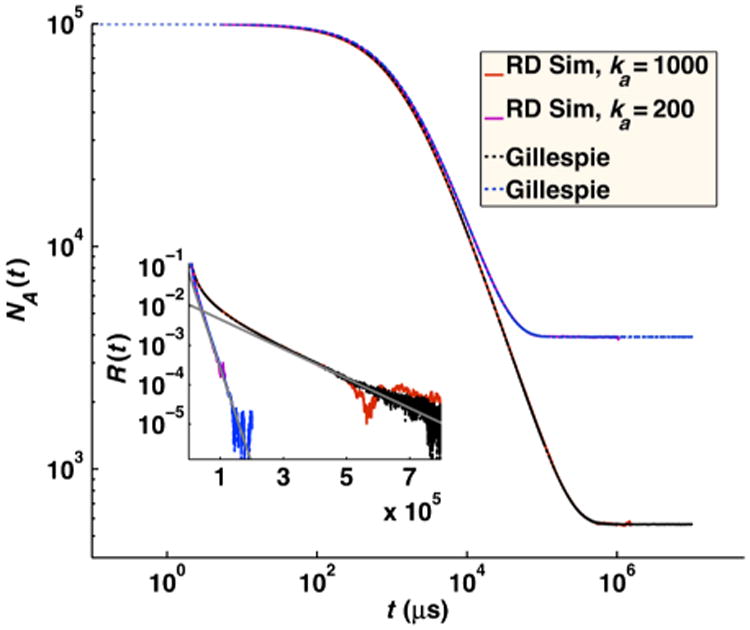
Simulations of reversible reactions *A + B*⇄ *C* with the FPR method (red and magenta) and with the Gillespie algorithm (black and blue). For both reactions, *A* and *B* particles are present at 52 *μ*M concentrations and each have *D* = 1 nm^2^/*μ*s with Δ*t* = 0.5 *μ*s. Reaction rates of *k_a_* = 1000 nm^3^/*μ*s, *k_b_* = 1 s^−1^ and *k_a_* = 200 nm^3^/*μ*s, *k_b_* = 10 s^−1^ are shown in red and magenta. The inset shows the relaxation *R*(*t*). The full relaxation is not known analytically, but is asymptotically exponential for well-mixed systems [*R*(*t*) ∼ exp{–*t*[*k*_on_(*A*_eq_ + *B*_eq_) + *k*_off_]}], represented by the solid gray lines with best-fit intercepts. The close agreement indicates that for these solution conditions, diffusion can accurately be represented as a constant contribution to the on rate. The transition to power-law relaxation for the RD systems happens at *R*(*t*) ∼ 10^−5^, beyond the statistical accuracy of these simulations.

**Fig. 12:**
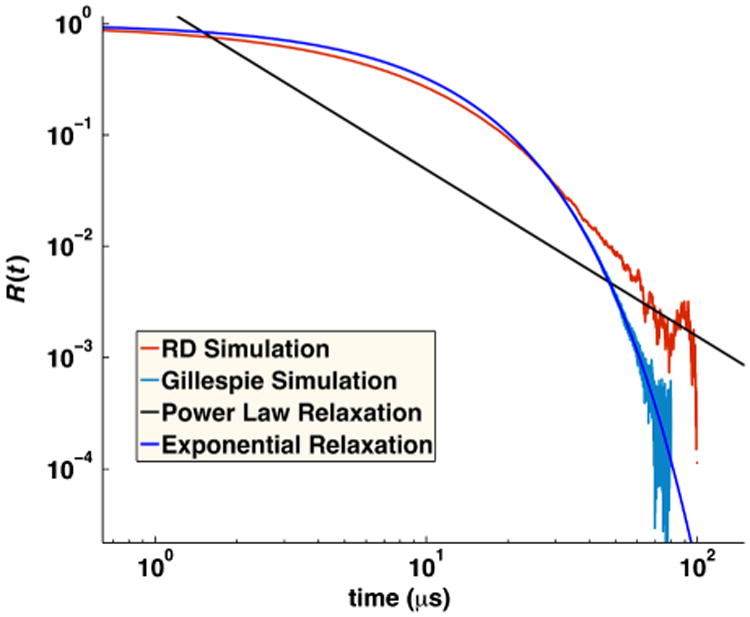
Reversible reactions with *k_a_* = 200 nm^3^/*μ*s and *k_b_* = 10^6^ s^−1^ and *A* and *B* at equal concentrations (52 *μ*M) with equal diffusion constants *D_A_* = D_B_ = 1 nm^2^/*μ*s and Δ*t* = 0.01 *μ*s. For these conditions, the behavior of the RD simulations (red) is clearly distinct from chemical kinetics (blue), with relaxation showing the power-law asymptotic relaxation (black) to equilibrium in contrast to the exponential relaxation of the homogeneous solutions (blue). The simulated *A*_eq_ value is here again used in the definition of *R* (*t*). The percent error of the simulated RD average to the exact A_eq_ is less than 1% (0.03%). The Gillespie trajectories are binned and averaged over 100 000 runs and the percent error of the Gillespie average is 0.0005%.

For solutions of reversibly reacting particles that are at comparable concentrations, the full time-dependent relaxation has not been determined analytically. Only the long-time asymptotics on approach to equilibrium have been shown to be exponential in time for chemical kinetics, and power law for RD systems [34,35]. In Fig. 11, we compare simulations of moderate concentrations of *A* and *B* particles that reversibly react. For these solution conditions, the results agree quite well with the behavior of chemical kinetics, solved using the Gillespie algorithm [36] using *k*_on_ and *k*_off_ defined above. This indicates that the effects of diffusion on the association rate over the time scales shown can accurately be absorbed into an overall rate *k*_on_ that accounts for both diffusion to contact and reaction at contact and equivalently for dissociation. The long-time asymptotics on approach to equilibrium for the RD system will eventually switch to obeying a power-law dependence on time. The switch to power-law relaxation occurs because spatial domains form as the system relaxes and the diffusion of particles to one another over these domains becomes the limiting step in the association between particles as equilibrium is approached. (Consequently, if the box is too small for domains to form, the relaxation will remain exponential.) For the solution conditions in Fig. 11, the simulation systems are not large enough to sensitively measure the onset of this power-law behavior, which begins at ∼0.15 s for *k_a_* = 200 nm^3^/*μ*s and at ∼1 s for *k_a_* = 1000 nm^3^/*μ*s, requiring high precision (10^−5^). Instead, in Fig. 12, we show simulations at the same moderate concentrations of *A* and *B* particles but with much faster equilibration (*k_b_* = 10^6^ s^−1^). For these conditions, the power-law asymptotic behavior derived in Ref. [34] is visible and contrasts with the exponential decay observed in chemical kinetics (solved using the Gillespie algorithm [36]).

In summary, the FPR algorithm reproduces the correct equilibrium and relaxation behavior for RD systems extremely well for both strongly and weakly interacting systems, with only small deviations for very dense and slow-moving systems [Fig. 10(a)]. For these systems, the accuracy can be improved by adjusting the association rate to correct for the undersampling of association that occurs with trajectory reweighting.

### C. Irreversible reactions with an interaction potential

We now consider particles that not only react with one another on collision, but also exert a force on one another. The Coulomb potential describes long-range repulsive or attractive interactions between molecules, and is a biologically reasonable addition to describing interactions between proteins at the coarse-grained level. We demonstrate the effects of adding both a repulsive and attractive Coulomb potential defined by *βV*(*r*) = (*r_c_/r*), where *r_c_* is >0 for a repulsive potential <0 for an attractive potential. The steady-state rate constant for this system is given by [31]

(31) $$k_{\text{on}}=4\pi D_{\text{tot}}r_{c}{\left[(1+\frac{4\pi D_{\text{tot}}r_{c}}{k_{a}})\exp(\frac{r_{c}}{\sigma })-1\right]}^{-1}.$$

If the potential were truncated beyond the reaction zone, this would alter the rate defined above, resulting in a higher rate for a truncated repulsive potential and a lower rate for a truncated attractive potential.

**Fig. 13:**
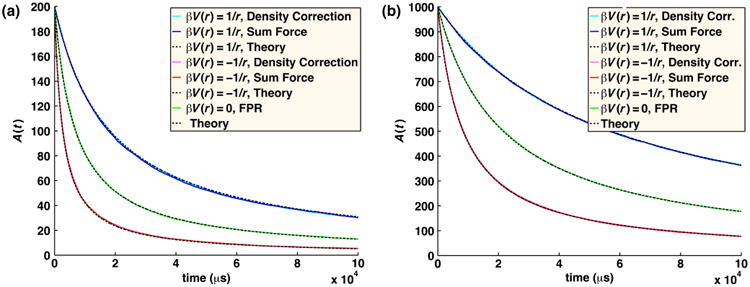
Irreversible reaction *A + A →* 0 with forces between particle pairs. (a) The *A* particles start at a concentration of 5.2 *μ*M and (b) 1.66 *μ*M. The reaction parameters are the same as in Fig. 7. The relaxation of *A*(*t*) is shown for these same reaction parameters in the presence of a repulsive potential (bluish solid curves), no potential (green solid curve), and an attractive potential (reddish solid curves). The black dashed lines represent the theoretical solution to Eq. (27) with *k*_on_ given by Eq. (31), which has values of *k*_on_ = 17.15, 46.31, and 119.57 nm^3^/*μ*s in that same order. Both long-range FPR approaches perform equally well at reproducing the association rate of the systems, although in the slightly smaller and more dense system in (a), the simulations trend to slightly faster association with the repulsive potential and slightly slower association with the attractive potential. Each curve is averaged over 100 trajectories.

In Fig. 13, we plot the solution to the irreversible association of *A* particles with one another given by Eq. (27) using *k*_on_ above, when *r*_c_ = 1 and −1, *k_a_* = 50 nm^3^/*μ*s, *D_A_* = 25 nm^2^/*μ*s (giving *D*_tot_ = 50 nm^2^/*μ*s), and Δt = 0.01 *μ*s. This time step is small enough that the single-step propagators have sufficient overlap. Trajectory overlap at long times is also not an issue, as the exit ratio distribution for these trajectories is similar to that in Fig. 4(a), tightly centered around 1. Interactions are summed using the minimum image convention, without using lattice sums. We compare the theoretical result with our many-body FPR simulations that have been adapted to account for the long-range forces by either (1) summing over all pairwise forces in the system for each particle or (2) correcting for the reweighting ratio when any pair first enters the reaction zone. The advantage of the first approach is that it will better maintain the density distribution of particles throughout the volume. The advantage of the second approach is that it does not require calculating all pairwise separations and is therefore substantially faster. Both approaches perform well in reproducing the association rates of particles in these many-body systems with an interaction potential.

## V. Discussion and Conclusions

We have developed a method for both accurately and efficiently modeling heterogeneous systems of reacting proteins at single-particle resolution. In contrast to the GFRD method [23], which is exact at the two-particle level, the FPR approach is approximate but is simpler to implement. Importantly, FPR scales readily to large and diverse reactant populations, and can adapt to changes to diffusion constants and reaction rates as a result of binding or reaction events. The FPR method has a rigorous theoretical foundation and provides an accurate numerical solution to the governing dynamical equations that correctly reproduces the behavior of rate-limited and diffusion-limited reactions, effectively eliminating the time-step dependence of more approximate methods [15]. The physical properties of the proteins modeled via diffusion constants, binding radii, and reaction rates are essentially independent of the simulation time step and numerical approach. The simplicity of the FPR method derives from the use of the free propagator to update particle positions, and the accuracy of the method stems from the use of the exact irreversible GF to evaluate reaction probabilities and to correct for the behavior of the free propagator through trajectory reweighting.

The FPR method can not only treat diffusing particles that react upon collision, but can also account for forces acting between particle pairs. In this way, it provides a unique resource for accurately capturing the effects of interaction potentials [24,37] on the kinetics of protein association. Introducing interaction potentials between proteins, such as the Coulomb potential studied here, accounts for an important physical factor controlling how proteins are steered to contact beyond just diffusional collisions. Coulombic interactions, in particular, are responsible for the fast association rates of the barnase-barstar complex [38]. The FPR method can incorporate potentials because the general framework derived in Sec. II B does not require the specific propagators defined in that section. The method can similarly be used for propagators that include not only free diffusion but rotation or orientational constraints [39]. Although these cases may produce extra challenges, requiring, for example, numerical calculation of the GF if it is nonanalytic, once the GFs are known, the many-particle simulations are still relatively simple to propagate. In particular, evaluating the numerical GF at a given separation is still substantially simpler than trying to sample positions from it, so the cost would likely still be lower than sampling positions from the exact propagators.

A concern when applying trajectory reweighting is the prospect of undersampling trajectories due to poor overlap between the exact and free propagators. Importantly, the weight factor is exactly one on average, which effectively ensures efficient sampling as long as weight distributions are not tail dominated. We find that tails become more dominant for very slow diffusion constants and dense systems (close to 1 mol/L), resulting in undersampling of certain trajectories. Fortunately, these conditions are not generally representative of biological systems, but they do serve as useful tests of the accuracy of the approach. Furthermore, we clearly establish that the source of the deviations in these systems is the undersampling of trajectories, and one can therefore anticipate that the deviations will decrease the apparent association rate. One can also use the condition of the weights averaging to exactly one as an indicator of a possible sampling issue. We present two solutions to correct for this issue, the simplest of which is to increase the reaction rate to compensate for the decrease in associating trajectories.

Here, we do not consider the effects of hydrodynamic interactions that establish long-range couplings in the diffusion of individual particles and can influence the dynamics and association rates of proteins [40-42]. In FPR, these hydrodynamic effects could be treated at least approximately by using particle-position updates with full hydrodynamic interactions, e.g., using the Ermak-McCammon integrator [43], combined with FPR path reweighting for simple Brownian dynamics to keep the weight factors analytically tractable.

Single-particle approaches to simulating large populations of proteins still lack much of the molecular and structural detail that truly defines the physical interactions between proteins. However, capturing atomistic or residue-level resolution in protein interaction dynamics is still far too costly to simulate and single-particle reaction diffusion offers opportunities to build in some of this detail by partitioning the protein surface to mimic separate interfaces or orientational constraints on binding. Methods that employ GFs, such as the one we derive here, have the potential to build in more microscopic detail to protein reactions and help bridge the gap between molecular models and particle-based models of protein interactions.
